# What is the Validity of Questionnaires Assessing Fruit and Vegetable Consumption in Children when Compared with Blood Biomarkers? A Meta-Analysis

**DOI:** 10.3390/nu10101396

**Published:** 2018-10-01

**Authors:** Tatiana S. Collese, Gabriela Vatavuk-Serrati, Marcus Vinicius Nascimento-Ferreira, Augusto César Ferreira De Moraes, Heráclito Barbosa Carvalho

**Affiliations:** YCARE (Youth/Child cArdiovascular Risk and Environmental) Research Group, Department of Preventive Medicine, Faculdade de Medicina, Universidade de Sao Paulo, Sao Paulo, SP 01246-903, Brazil; gabrielavserrati@gmail.com (G.V.-S.); marcus1986@usp.br (M.V.N.-F.); augustocesar.demoraes@usp.br (A.C.F.D.M.); heracc@usp.br (H.B.C.)

**Keywords:** systematic review, meta-analysis, children, fruits and vegetables, biomarkers, vitamins, validity

## Abstract

Fruit and vegetable consumption has been associated with improved health outcomes in children. As an extensive number of questionnaires are currently used to assess fruit and vegetable consumption, we performed a systematic review of the criterion validity of questionnaires used to estimate fruit and vegetable consumption in children, considering blood biomarkers as the reference method. Five electronic databases (MEDLINE, CINAHL, Scopus, PsycINFO, Web of Science) were searched from database inception to 23 July 2018. The search strategy used the following sets of descriptors: children; fruits and vegetables; dietary questionnaires; blood biomarkers; and validation coefficient. The search terms were adapted for use with other databases in combination with database-specific filters. Potentially eligible articles were selected independently by two reviewers, separately, following the Preferred Reporting Items for Systematic Reviews and Meta-Analyses (PRISMA) guidelines. Two articles meeting the inclusion criteria were included. The main reason for study exclusion was the sample age range, which included adolescents. The pooled correlation coefficient was 0.32 (95% confidence interval: 0.24–0.40).This review provided insights into assessment methods of fruit and vegetable consumption in children. Although further studies are required, questionnaires for assessing fruit and vegetable consumption have fair criterion validity in children.

## 1. Introduction

International health associations recommend that children have a diet rich in vegetables, fruits, whole grains, low-fat dairy products, legumes, fish, and lean meat, and low in saturated fat, trans fat, and cholesterol, to help maintain a healthy weight and promote cardiovascular health [1]. Given the role that the diet plays in children’s health, it is crucial to accurately determine their dietary intake and to estimate the relationship between the foods consumed and nutrients.

Regular fruit and vegetable consumption is routinely suggested as a key component of promoting health because they are an important source of nutrients, such as water, fiber, potassium, folic acid, vitamins, and phytochemicals [1,2]. Several systematic reviews and meta-analyses have found consistent associations between fruit and vegetable consumption and lower risk of cardiovascular disease, diabetes, cancer, and other chronic diseases [3,4,5]. Despite the well-known benefits of consuming fruits and vegetables, children are not meeting the recommended level of five servings per day [6]. Consequently, increasing fruit and vegetable consumption in children is one of the major goals of dietary interventions, worldwide [6]. Thus, valid questionnaires to assess fruit and vegetable consumption are essential to identify eating habit changes in response to interventions, and to analyze the impact of fruit consumption on health [7].

In nutritional epidemiology, the most used methods to estimate fruit and vegetable intake are subjective instruments, such as food frequency questionnaires (FFQs) and dietary recalls, because they are simple, cost-effective methods to assess long-term eating habits for large samples [8,9]. However, these subjective instruments are susceptible to both random and systematic errors [10,11], especially when applied to children, due to their limited skills to report their own dietary intake, depending on parental (or whoever is responsible for their food) literacy and motivation to complete these questionnaires [12].

As all dietary assessment methods (e.g., 24 h recalls, FFQs, smart-phone apps) have their own strengths and limitations, dietary questionnaires are usually validated against an objective measurement, such as blood biomarkers or doubly-labeled water [8,13]. Previous studies proposed that plasma vitamins, such as vitamin A, E, and C, are a reliable concentration biomarker of usual fruit and vegetable intake. Hence they are commonly used in validity studies of dietary assessment methods as independent proxy measures of fruit and vegetable consumption [14], and to evaluate whether sources of random error are independent of errors associated with measurement by questionnaire and/or inaccuracies within nutrient databases [15].

However, these plasma vitamins are still relatively recent, and a significant amount of studies are warranted in nutritional epidemiology [16]. It concerns deciphering whether these biomarkers are markers of dietary consumption or markers of altered metabolism, as a result of the food intake [16], or regarding the individual variability in absorption or availability [17]. Validation studies with blood biomarkers have been carried out in adults [10,11], but relatively few studies were conducted in children, lacking literature on key issues such as the impact of the period between fruit and vegetable consumption and the blood biomarker analysis, or the stability of blood biomarkers in children aged 3 years to less than 10 years.

Therefore, the research question of this review was “What is the criteria validity of questionnaires used to estimate fruit and vegetable consumption in children when compared with blood biomarkers?”

## 2. Methods

This systematic review was designed and conducted according to the guidelines of the Preferred Reporting Items for Systematic Reviews and Meta-Analyses (PRISMA) checklist (Supplementary Material 1), as described in Reference [18]. At study initiation, no similar systematic reviews were available in PROSPERO (International Prospective Register of Systematic Reviews) or the following databases: Biomed Central, Medical Literature Analysis and Retrieval System Online (MEDLINE), Web of Science, Cumulative Index to Nursing and Allied Health Literature (CINAHL), Scopus, and PsycINFO. Thus, this protocol was registered in PROSPERO (CRD42017072531).

### 2.1. Study Selection

Five electronic databases—MEDLINE, CINAHL, Scopus, PsycINFO, and Web of Science—were searched from inception to 20 December 2017. The searches were registered in the National Center for Biotechnology Information (US National Library of Medicine, Bethesda, MD, USA), so that continual updates on new publications would be received until 23 July 2018, and the searches were updated until this same date. References of selected articles were analyzed, and the corresponding authors were contacted to identify other relevant studies. The descriptors and medical terms used for the database search were as follows: *(Child OR Children OR school OR boy OR son OR girl OR daughter) AND (Fruit OR Vegetable) AND (Questionnaire OR 24h Dietary Recall OR 24 h-Dietary-Recall OR 24 h Dietary Recall OR Recall OR Food Frequency Questionnaire OR FFQ) AND (Biomarker OR Blood OR concentration biomarker OR serum OR serum marker OR carotenoids OR vitamins) AND (Validation OR Validity OR coefficient OR reliability).*

Two investigators examined the articles and performed the data screening, the data extraction, and the quality assessment independently. Inter-reviewer discrepancies were resolved by consensus, and a third reviewer was consulted for unresolved discrepancies. Relevant articles were obtained in full and were assessed for eligibility and exclusion criteria.

### 2.2. Eligibility Criteria

As the goal of this systematic review was to assess evidence, specifically for validation studies of questionnaires used to estimate fruit and vegetable consumption in children when compared with blood biomarkers, this type of review must clearly delineate the population in question. Consequently, the results may apply only to school-aged children.

The literature suggests that children are already able to help their parents to report their food intake with relative accuracy [19]. However, after 10 years of age, the World Health Organization [20] classifies the population as adolescent, with more independence and skills, which strongly influence decision making, peer affiliation, and behavior [21], allowing them to decide on and self-report their own dietary intake. In this sense, we considered only the children between three years of age until nine years and 11 months, as presented in Table 1.

#### Exclusion Criteria

Studies where the participants had different diseases (or disturbances) that could interfere with fruit and vegetable consumption were excluded. These criteria were set to increase inter-study comparability. In addition, review articles, conference papers, or books were excluded.

### 2.3. Data Screening and Extraction

Potentially eligible articles were selected for inclusion according to the following sequence:

1. Articles published in English, Spanish, and Portuguese were identified;
2. Titles of the articles were screened;
3. Abstracts were screened; and
4. The full texts of the articles were reviewed to determine whether they met the inclusion criteria.

Articles were saved in the EndNote Web reference manager software version 3.1 (Thomson Research Software, Carlsbad, CA, USA), and duplicates were removed. The data extracted included the author, title of the study, citation and contact details, and study eligibility (Table 1). When studies did not meet the eligibility criteria, the main reason for exclusion was documented in ink. Thereafter, a table (Supplementary Material 2) was created by each reviewer with additional details about the study, such as the sample size, settings, exposure/outcome definition and unit of measurement, statistical analysis, bias (funding sources, adjustments), key conclusions, authors, miscellaneous comments from the study authors, references to other relevant studies, limitations, correspondence required, and miscellaneous comments by the authors reviewing the included studies.

### 2.4. Quality Assessment

All retrieved articles were independently assessed and critically appraised using the standardized checklist of the STROBE-nut (adapted to our review) to identify sources of bias, performance, attrition, and detection. Data relevant to this review included the study design, characteristics of the research subjects, dietary assessment methods used, results, discussion, bias assessment, and ethics information.

### 2.5. Strategy for Data Synthesis

The reporting status of fruit and vegetable consumption in each of the included studies were determined from that listed within the results section of the included articles. The reporting status of each study was determined using three predefined categories. The categories were dependent on the level of accuracy of reported fruit and vegetable consumption, compared to measured blood biomarkers. When available from included studies, agreement estimates were extracted if the reporting status of research subjects correlated to various characteristics of the group. These characteristics included demographic statistics (age and sex), anthropometric characteristics (height, weight, and body mass index), and the study design. Limitations of each study and the evidence level were recorded as well. The agreement estimates from study questionnaires were synthesized.

### 2.6. Statistics

We used the Stata 14 (Stata Corp., College Station, TX, USA) program for all statistical analyses. Our outcomes were the correlation coefficients between the questionnaire and blood biomarkers, which we calculated with their corresponding 95% confidence intervals (considering *p* < 0.05 as significance level). In addition, we calculated the sample-weighted pooled correlation coefficient for the meta-analysis with the random-effects model, due to the moderate heterogeneity found in each study. We estimated the strength of the agreement of the Spearman rank correlation coefficient cutoff points using the classification for dietary assessment methods: weak < 0.20, fair = 0.21 to 0.40, moderate = 0.41 to 0.60, good = 0.61 to 0.80, and >0.80, very good [22]. Additionally, we verified the heterogeneity of the studies using the *I-squared* test (percentages and *p* values under 0.05 were considered significant). Heterogeneity levels of 25%, 50%, and 75% were considered low, moderate, and high, respectively [23]. We performed the Egger’s regression test to verify potential publication bias (positive or negative results) and small-study effects. We also generated the funnel plot (Supplementary Material 3) to examine the potential bias graphically.

## 3. Results

**Figure 1:**
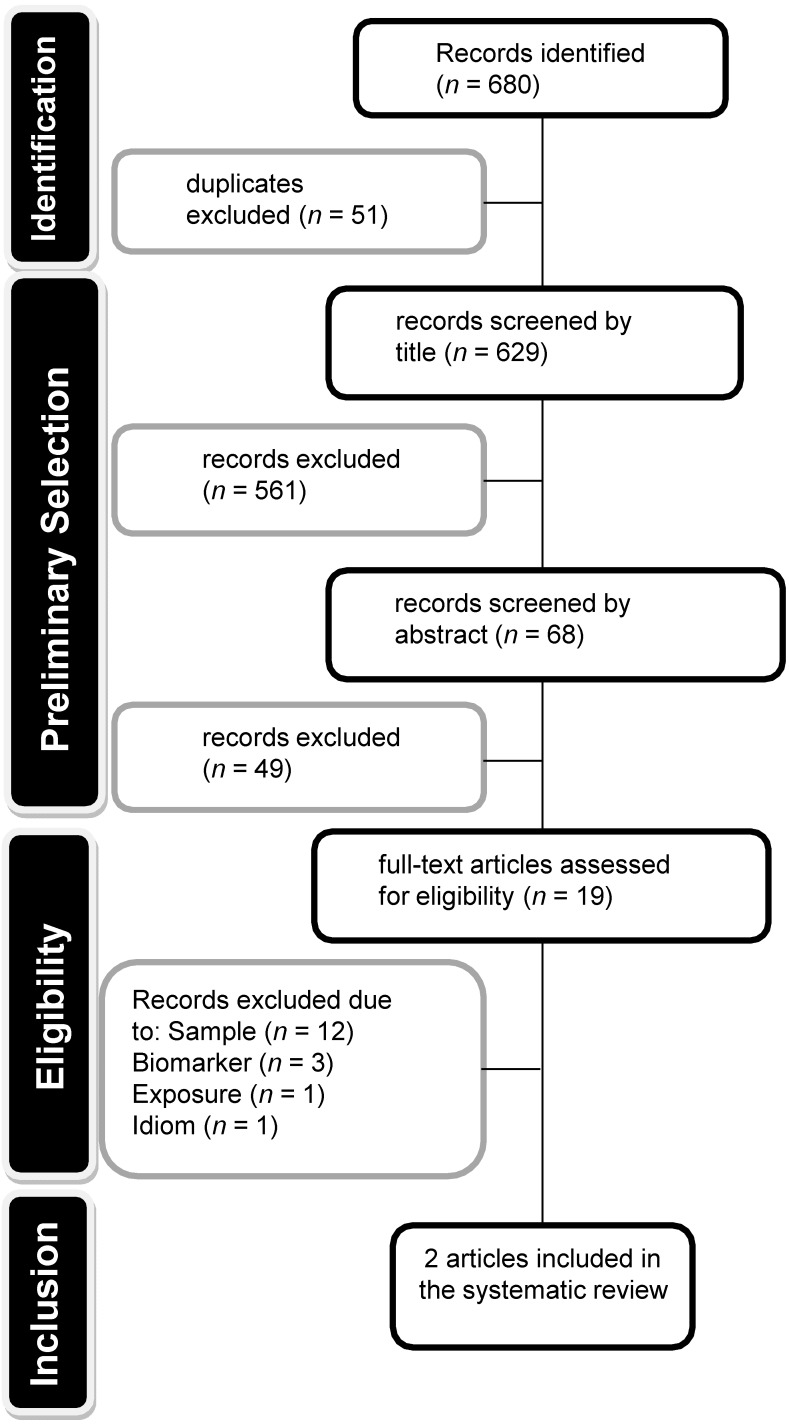
Flow chart of article identification, retrieval, and inclusion, for the systematic review.

The literature search provided 680 potentially eligible records: 4 from PsycINFO, 42 from Embase, 71 from Web of Science, 430 from Scopus, and 133 from Medline (see Appendix 1 in the Supporting Information online). After duplicates (*n* = 51) were excluded, 629 studies remained and were screened by title and, posteriorly, by abstract, according to the established inclusion criteria, resulting in 19 records for which the full-text was assessed for eligibility. Of those, the main reasons for exclusion were the population (studies included participants over 10 years of age) and biomarker (such as skin carotenoids), as shown in Figure 1. More details about these 19 study articles are stated in Supplementary Material 4. Consequently, two studies were eligible and were included in this systematic review.

The two final eligible articles included in this systematic review were published in English, but they are from different countries, one from Europe and the other from North America (Table 2). We found one study published in 1993 [24], but Medin et al. [25] was more recent, published in 2016.

Table 3 shows that both studies asked for the parental report; however, Byers et al. [24] used the FFQ regarding the previous three months and considered fruit juice together with fruit and vegetable consumption. Medin et al. [25] used a web-based food recall, for four consecutive nights, but they assessed only vegetable intake for 4th grade children (8–9 years of age).

Nine different types of biomarkers were used to validate fruit and vegetable consumption. Both studies analyzed carotenoids (α-carotene, β-carotene, and cryptoxanthin), but Medin et al. [25] also included lycopene, lutein, and zeaxanthin in the sum of total carotenoids. On the other hand, Byers et al. [24] analyzed vitamin A (retinol), vitamin E (α-tocopherol), and vitamin C (ascorbic acid). High-performance liquid chromatography (HPLC), which is considered the gold standard analytical technique for characterization and analysis of carotenoids in biological and food samples [26], was used to assess plasma carotenoids in all studies (and for vitamin E in Byers et al. [24]), and spectrophotometry was used to assess plasma vitamin C (Table 3).

The correlation coefficients between fruit and vegetable consumption and blood biomarkers are presented in Table 4. The validity coefficients were fair to moderate, varying from 0.24 (for vitamin E) to 0.47 (carotenoids). Both studies calculated the Spearman rank correlation coefficients and adjusted the analyses for age, sex, ethnicity, and body mass index.

**Figure 2:**
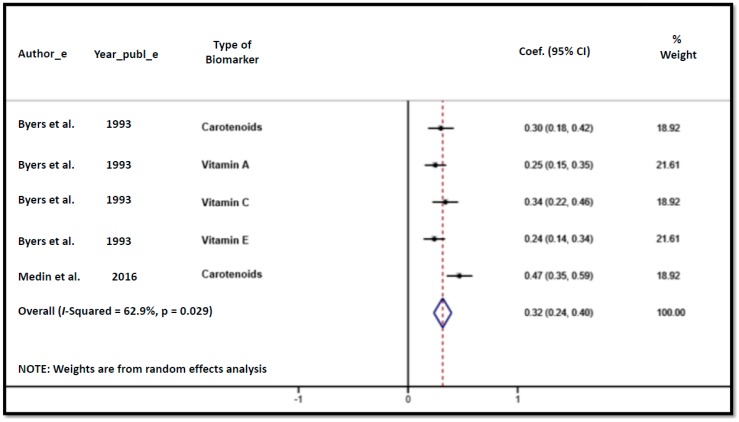
Forest-plot for correlation coefficients of fruit and vegetable consumption assessed by questionnaires and blood biomarkers.

The heterogeneity between studies was moderate (I-squared = 62.9%, *p* = 0.029), and the sample-weighted pooled correlation coefficient for biomarkers was fair (*r* = 0.32, 95% CI: 0.24–0.40), as shown in the Figure 2. We were not able to stratify the analysis into subgroup categories due to the small number of eligible studies included in the systematic review.

## 4. Discussion

At present, there is no strong evidence regarding the validity of questionnaires for assessing fruit and vegetable consumption in children with blood biomarkers. Thus, we conducted a comprehensive systematic review of validity studies of questionnaires to assess fruit and vegetable consumption in children in comparison with blood biomarkers. Our findings indicated that this criteria validity was fair. This validity stems from the challenges inherent to dietary assessment through questionnaires, in addition to the fact that in children, we should consider the parent report, which is the subjective perception of the parents about their children’s feeding habits [13,27].

The limited number studies (*n* = 2) meeting the inclusion criteria was likely due to the lack of validated dietary questionnaires, maybe because conducting blood collection in school children is less feasible, for practical and financial reasons [28], and due to the sample age range, since different age group definitions were used for children (e.g., together with infants or adolescents). For example, the study of Biltoft-Jensen et al. [29], which fulfilled our eligibility criteria, except for the population age range, which was between 8 and 11 years of age. It is correct that 11-year-old individuals are still children; however, the timing of biological maturation clearly signals entry into adolescence [21], and with more cognitive abilities that enable the researchers to apply the self-report of fruit and vegetable consumption. Therefore, it was excluded from our review.

It is important to emphasize that the credibility of our findings does not rely on the number of studies included in the meta-analysis (*n* = 2), as we conducted an exhaustive and reproducible literature search to produce results with the highest level of quality of evidence available. Murad et al. [30] state that a systematic review or meta-analysis should evaluate the credibility of the methods applied on the systematic review, and that this credibility depends on whether the review addressed a specific and objective question; included an exhaustive literature search; clearly indicated the reproducibility of the selection and assessment of studies; and declared the results in a convenient way that enables future research development. Furthermore, even though only two studies were eligible in our systematic review, each biomarker was considered separately in the analysis; in other words, we evaluated five different items (*n* = 5).

The sample-weighted pooled correlation coefficient found in our study (*r* = 0.32, 95% CI: 0.24–0.40) was fair, and comparisons with other validity studies carried out in children and adolescents (9–12 years) showed similar Pearson correlation coefficients for vegetable (0.26, *p* = 0.13) and fruit intake (0.49; *p* = 0.003) with total plasma carotenoids [31]. Studies carried out in adults showed a slight increase in the Spearman correlation coefficients for alpha-carotene (0.42; CI: 0.09–0.96), beta-carotene (0.40; CI: 0.09–0.99), and total plasma carotenoids (0.51; CI: 0.12–1.00) [32]. However, these correlations are not so distant from our results, and it is relevant to note that their confidence intervals were broad.

### 4.1. Dietary Assessment Methods

Measuring fruit and vegetable consumption is complex, with interactions of different food compounds, cultural, and socioeconomic conditions, in variable proportions [10,33]. Until this moment, an ideal questionnaire to assess fruit and vegetable consumption is unknown [28]. There is an extensive number of methods to perform this evaluation; however, all of them have both random and systematic limitations and errors [10].

It is difficult to determine from the studies included in this review who is the most accurate reporter of a child’s fruit and vegetable intake, and which method is most accurate and reliable. It is important to note that mere participation in a research study may have biased the data reported for each child, because parents may have selectively reported higher consumption of fruit and vegetable of their child due to their involvement in the study. Reporting methods also depend on the difficulties associated with the method of reporting, and with the parental educational level.

### 4.2. Food Frequency Questionnaires

Byers et al. [24] used an FFQ to estimate fruit and vegetable consumption in children. Some advantages of the use of the FFQ are as follows: it can be given to the children’s parents with limited explanation; it is a good method to obtain data regarding food eaten less than twice a week; questions can be included to allow different portion sizes (adjusted); and the food/nutrient database only needs to cover commonly eaten foods [34].

Disadvantages: it is time consuming; parents may find it difficult to decide how often their child eats fruit or vegetables; the FFQ often underestimates foods eaten regularly or in large quantities if the portion sizes used in the questionnaire are based on average intake; if a portion size is specified in the FFQ, parents may find it difficult to interpret and answer the question meaningfully; and furthermore, it depends on the level of literacy of the parents [34].

### 4.3. Web-Based Food Recall

Medin et al. [25] showed a higher correlation coefficient (0.47) for vegetable consumption, using a web-based food recall for four consecutive nights. The development of technology-based research tools for the assessment of intake, such as the web-based food recalls, has the potential to reach large populations and reduce language barriers through the use of images rather than verbal descriptions [19]. Technology allows for greater scale and efficiencies for researchers to rely on regular dietary intake data, for surveillance and monitoring [19]. The conversion of paper-based methods into web-based methods may have benefits, including faster completion, greater reach, and the ability to maximize the collection of complete data [19], which might explain part of the higher correlation coefficient found by Medin et al. [25], when compared to other studies that used the FFQ. Furthermore, two small details might have made a great difference in the Medin et al. [25] results: participants were given a personal gift card as an incentive for participating in the study, which may have changed their engagement in it; a voice-assisted cartoon character helped the children and parents to complete the questionnaires, with pop-up reminders to reduce the problem of food omissions, and in a more friendly way for kids—this was shown to be a promising tool for application in pediatric populations.

In addition, Medin et al. [25] included four nightly 24-h recalls. This period is more than the literature recommendation (three recalls per person) [34], and since parents answered the questionnaire at night (the end of the day), it was easier for them to remember their child’s fruit and vegetable consumption on the same day, minimizing the memory bias as well [34].

However, web-based food recalls reduce the limitations in terms of misreporting. Thus, there is a need for continued development of methods, as well as to continue to evolve statistical methods to mitigate error. Training researchers, clinical staff, and research subjects on the use of the technology and the method is still highly warranted and might improve results and compliance with the dietary method [19].

To be confident about the fruit and vegetable data collected, one should combine the FFQ with food recall methods, trying to ascertain both frequent and infrequent fruit and vegetable consumption [35]. Additionally, if possible, combining technology with both dietary assessment methods is a good alternative to reduce the burden of data handling and to make this process more friendly and joyful.

### 4.4. Creativity

**Figure 3:**
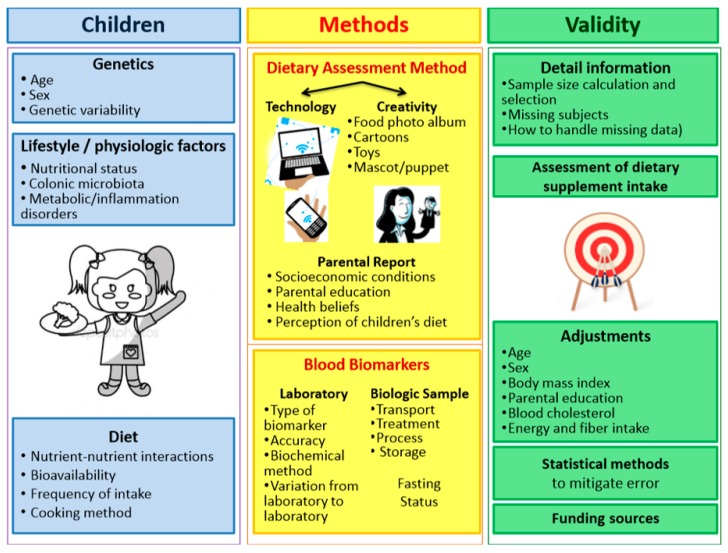
Main factors regarding the validity of dietary assessment methods in children.

The integration of technology, pictures, drawings, cartoons, and puppets into traditional dietary questionnaires, considering the main factors inherent (Figure 3) to this approach, might provide additional insights to mitigate errors in the fruit and vegetable assessment.

When financial resources are scarce, like in studies conducted in low and middle-income countries, using a bit of creativity might be a good solution. For example, if it is not possible to combine the technology in the process of dietary assessment, a simple art-based method like a “mascot” (drawn on paper, as a cartoon, as a toy or a hand puppet) can make a great difference in the motivation and involvement to answer the questionnaire, turning this process into a fun moment for kids [36,37]. Additionally, pictorial assessment, such as the assessment with the help of food photography albums, food drawings or food replicas (three-dimensional models), might also be a cheaper and smart alternative to enhance motivation and give visual support to estimate fruit and vegetable portion sizes [38,39].

### 4.5. Administration Mode

A recent systematic review of the validity of dietary assessment methods in children when compared with doubly labeled water suggests that using a parental reporter is the most accurate method for reporting dietary intake in children aged 4–11 years [13]. However, it should be noted that parents were the proxy reporters for the children; thus, some bias can be involved in this report. For example, the parental weight status may influence the accuracy of their report, as observed in a study with obese parents, who may underestimate the food intake of their obese children [40].

### 4.6. Blood Biomarkers

It is important to bear in mind that there are issues pertaining to bio accessibility and bioavailability of vitamin A, E, and C in fruits and vegetables, depending on several factors, such as genotype, ripening time, cultivation method, and climatic conditions, processing, food source, food particle size and location, cooking method, and the content of fiber of the fruit or vegetable [41,42]. Moreover, different parts of the same plant may also contain different types and amounts of vitamins [41].

In addition, knowing that most vitamins have a half-life of one or 2 months [43], it is difficult to assess their blood availability and to compare it over long previous periods, such as Byers et al. [24] did with their FFQ. Blood vitamins cannot be translated into absolute levels of fruit and vegetable consumption, but these biomarkers do correlate with fruit and vegetable consumption in certain strengths, and they are commonly applied in dietary assessment studies [28].

There are other factors relating to the children that may also affect the measurement and utility of these biomarkers to properly reflect their fruit and vegetable consumption. For example, genetic variability, lifestyle or physiologic factors (e.g., colonic microbiota, body mass index, and metabolic or inflammation disorders) [44], dietary factors (e.g., frequency of intake and nutrient–nutrient interactions), blood sample (e.g., whole blood, plasma, serum, conditions of sample collection, transport, treatment, process, and storage), fasting status before examination (which neither of the studies required), and laboratory analytical methodology (e.g., accuracy, detection of limits, variation from laboratory to laboratory) [28].

Even with all these factors influencing vitamins blood levels, this method is still considered by the scientific community as a reference method for fruit and vegetable consumption in epidemiological and clinical studies.

### 4.7. Adjustment for Confounders

In addition, appropriate adjustments for confounding variables are critical for understanding how factors of interest are interrelated within a population, as there may be a range of plausible measurement errors that have a slight influence on these associations [45]. The two eligible studies reported adjusting for confounders; however, the variables in the adjusted model differed according to the study. Hence, we suggest that future studies should consider, at least, the sex, age, body mass index, parental education, blood cholesterol, and energy and fiber intake to be included in the fully-adjusted model.

### 4.8. Methodological Quality

To the best of our knowledge, there is no evidence regarding the validity of questionnaires for assessing fruit and vegetable consumption in children with blood biomarkers. However, other systematic reviews addressing the validity of dietary assessment questionnaires and objective methods, such as double label water [13] or skin biomarkers [19], have consistently reported low methodological quality of the studies as well. Other studies in the field of physical activity in children, validating questionnaires with objective methods (e.g., accelerometers), also reported low methodological quality, especially regarding the absence of information about missing subjects and how the missing data were handled [46].

In this review, only two studies were included. Based on the STROBE checklist, the major bias identified was related to the information bias, particularly due to the heterogeneity between studies in the applied methodologies. The absence of information about how the study size was calculated, or how missing data were handled was a flaw in both studies. Byers et al. [24] did not declare any adjustment for parental education level, nor information on whether fruit and vegetable intake was reported with or without the inclusion of dietary supplement intake, nor about funding sources that could influence their results.

### 4.9. Bias

The findings of our review indicated that more validity studies of fruit and vegetable consumption need to be carried out in school-aged children. The two eligible studies were published in English and in high-income countries. Therefore, it indicates a potential risk of publication bias, which can practically be explained by the expensive cost of conducting a validity study with blood biomarkers [28].

Moreover, most of the 19 remaining full-text articles assessed for eligibility used the plasma carotenes as a reference method, but only two articles assessed vitamin E [24,47], and one article assessed vitamin C [24]. This bias is also probably related to the costs of vitamin C, to its very sensitive biochemical methodology, and to the many difficulties and bureaucratic barriers for achieving the reactants necessary for this methodology.

As dietary assessment cannot be estimated without error, it is paramount to attempt to understand their effect on the data collected to minimize the confounding effect [17].

Byers et al. studied a sample of parents from a selective group of volunteers who reported their children’s food intake once a year. Thus, the validity of their reports was probably higher than other parents, because their motivation, involvement, and knowledge in the next step of the study could be different to other parents who have never been involved in a nutritional research [24].

Another relevant issue of concern is the social approval of fruits and vegetables. As they are widely known as healthy foods [48], parents may have reported their children’s fruit and vegetable consumption, either consciously or subconsciously, in ways that make them appear favorable to the researchers [17].

### 4.10. Heterogeneity between Studies in the Meta-Analysis

A moderate degree of heterogeneity was found in our meta-analyses (*I*^2^ = 62.9%, *p* < 0.029). At first, we found a higher degree of heterogeneity in our meta-analyses (*I*^2^ = 94.1%, *p* < 0.001), and it was more consistent with other systematic reviews regarding dietary assessment [9,19]. However, after critical point-by-point reanalysisin each article, we decided that some characteristics were not very specific to our eligibility criteria. For example, the study of Biltoft-Jensen et al. [29] that included the 11-year-old adolescents in the analysis and applied a self-report administration of the questionnaire; or the study of Royo-Bordonada et al. [47] that analyzed fruit and vegetable consumption as part of the dietary variety index (together with sausage and other foods that contributed to the dietary variety). Consequently, we had to exclude these two studies from the meta-analysis, since they had important sources of heterogeneity and did not fulfill our eligibility criteria [49]. After this exclusion, the remaining heterogeneity could be partially explained by the characteristics inherent to the different types of questionnaires used (e.g., recall period, number of items, recording bias, web-based system) and the biomarker (e.g., type of biomarker analyzed, duration of measure, different cutoff points).

### 4.11. Strengths and Limitations

The strengths of the current review included the use of a comprehensive search strategy and data collected from children aged 3 years to less than 10 years. To the best of our knowledge, this is the first attempt to provide estimates of the true effect sizes for assessing fruit and vegetable consumption, and to conduct a meta-analysis. Additionally, screening was performed by two independent authors; searches were updated and included papers written not only in English, but also in Spanish and Portuguese. The PRISMA statement was adopted, and a methodological quality rating was performed separately, to assist in interpreting the findings. The strength of this review was that two independent authors conducted the data extraction and the methodological quality assessment.

Despite these strengths, limitations should be noted. Although a comprehensive search was conducted, the agreement analyses were restricted to information from electronically published data, to ensure the reproducibility of the results.

Even though we considered Spanish and Portuguese for the literature search and screening, the two eligible articles were published in English. Both dietary habits and parental awareness can change considerably as children age; therefore, these findings may not be generalized to children of other ages. Moreover, although the dietary methods applied in each study were different, both methods are largely used in nutritional epidemiology [50] and in studies regarding fruit and vegetable consumption by children [51], enabling the meta-analysis, once we defined the correlation coefficients of these methods as our outcome [30].

## 5. Conclusions

Our findings suggest that the criterion validity of questionnaires and blood biomarkers for assessing fruit and vegetable consumption in children is fair. The assessment of food consumption—especially fruit and vegetable—in children still faces many challenges. However, having a thorough understanding of the main factors that may influence this assessment can be a promising path to enhance the validity of dietary assessment methods. Since measurement error is a key remaining difficulty, validity studies of questionnaires to assess fruit and vegetable consumption should be conducted with caution and interpreted within the broad context of the field. For example, careful study design and meticulous development of the dietary assessment tools can be validated with blood biomarkers, as long as the whole process of blood collection and analysis is adequate and rigorously controlled. Moreover, informed statistical analysis, with appropriate adjustments, can reduce the impact of these errors.

Finally, the incorporation of creativity and the development of technology, despite the challenges, can lead to advancements in the field of dietary assessment methods, and therefore, have an important role in the promising future of nutritional epidemiology.

## Figures and Tables

**Table 1:** Eligibility criteria included in the systematic review.

Eligibility Criteria	
Population	Children (3 years to less than 10 years of age) in a free-living environment, without any specific condition (e.g., eating disorder, obesity, diabetes) or children under medical care.
Exposure	Fruit and/or vegetable consumption estimated by questionnaire.
Comparator	Blood biomarkers for fruit and/or vegetable consumption.
Outcome	Validation coefficients of fruit and/or vegetable consumption.
Study	Original articles.

**Table 2:** Description of the included studies.

Reference	Survey Year	Country	Study/Article Title	Sample Age (Years)	Sample Size		Study Type
					Total (*n*)	Girls (%)	
Byers et al. (1993) [24]	1990	USA	The accuracy of parental reports of their children’s intake of fruits and vegetables: Validation of a food frequency questionnaire with serum levels of carotenoids and vitamins C, A, and E.	06 to 10	97	55.7	Validity
Medin et al. (2016) [25]	2013	Norway	Associations between reported intakes of carotenoid-rich foods and concentrations of carotenoids in plasma: A validation study of a web-based food recall for children and adolescents.	08 to 09 12 to 14	121	55.6	Validity

**Table 3:** Validity studies on questionnaires to assess fruit and vegetable consumption with blood biomarkers in children.

Reference	Exposure Assessment Method	Period of Report	Report	Consumption	Supplements Assessed	Nutritional Database	Blood Biomarkers	Biochemical Method	Fasting Time
Byers et al. (1993) [24]	FFQ (111 food items)	Previous 3 months	Parental	Median fruits, fruit juice, and vegetables Girls: 3.7 Boys: 2.9 (serving/day)	Not declared	Willet	α-Carotene β-Carotene Cryptoxanthin α-tocopherol Ascorbic Acid	HLPC (carotenoids, vitamin A and E), Spectrophotometry (vitamin C)	Not required
Medin et al. (2016) [25]	Web-based food recall	4 consecutive nights	Parental	Median fruits, fruit juice, and vegetables: 225.1 (g/day)	Yes + supplements containing carotenoids	USDA National Nutrient Database	α-Carotene β-Carotene β-Cryptoxanthin Lycopene Lutein Zeaxanthin	HPLC	Non-fasting

Abbreviations: FFQ: food frequency questionnaire; HPLC: high-performance liquid chromatography.

**Table 4:** Outcome of the included studies.

Reference	Correlations between diet and Blood				Criterion Validity	Covariates in Fully Adjusted Model	Conflict of Interest
	Vitamin A		Vitamin E	Vitamin C			
	Carotenoids	Retinol					
Byers et al. (1993) [24]	0.30	0.25	0.24	0.34	Spearman	Age, sex, ethnicity, family history of coronary artery disease, BMI, TG, serum total cholesterol, total caloric intake	Not declared
Medin et al. (2016) [25]	Vegetable intake: 0.47	-	-	-	Spearman	Age, sex, ethnicity, family structure, BMI, parental education level	None

Abbreviations: BMI: body mass index; TG: serum triglycerides.

## Supplementary Materials

The following are available online at http://www.mdpi.com/2072-6643/10/10/1396/s1, Supplementary Material 1: PRISMA checklist; Supplementary Material 2: Extraction form of full-text articles assessed for eligibility; Supplementary Material 3: Funnel-plot for correlation coefficients of fruit and vegetable consumption assessed by questionnaires and blood biomarkers; Supplementary Material 4: Main reasons for exclusion of the full-text articles assessed for eligibility.
