# Supplementary material for: What is the Validity of Questionnaires Assessing Fruit and Vegetable Consumption in Children when Compared with Blood Biomarkers? A Meta-Analysis
Source: Nutrients. 2018 Oct 1;10(10):1396. doi: 10.3390/nu10101396 (PMC6212808; doi:10.3390/nu10101396)
Supplement: Supplementary file 1 [file nutrients-10-01396-s001.zip › Additional file 2.pdf]

Supplementary Material 2 Extraction form of full-text articles assessed for eligibility.

**Reference**	**Byers et al. (1993)**	**Royo-Bordonada et al. (2003)**	**Biltoft-Jensen et al. (2013)**	**Aguilar et al. (2014)**	**Augusto et al. (2014)**	**Medin et al. (2016)**
**Survey Year**	1985-1990	1998-1999	2011	--	2007	2013
**Country**	USA	Spain	Denmark	USA	Brazil	Norway
**Article title**	The accuracy of parental reports of their children's intake of fruits and vegetables: Validation of a food frequency questionnaire with serum levels of carotenoids and vitamins C, A, and E	Greater dietary variety is associated with better biochemical nutritional status in Spanish children: The Four Provinces Study	Evaluation of Web-based Dietary Assessment Software for Children: comparing reported fruit, juice and vegetable intakes with plasma carotenoid concentration and school lunch observations	Skin carotenoids: A biomarker of fruit and vegetable consumption in children	Associations between low consumption of fruits and vegetables and nutritional deficiencies in Brazilian schoolchildren	Associations between reported intakes of carotenoid-rich foods and concentrations of carotenoids in plasma: A validation study of a web-based food recall for children and adolescents
**Sample age (years)**	06 to 10	06 to 07	08 to **11 (ADOLESCENT)**	05 to **17 (ADOLESCENT)**	04 to 10 **(PRE-SCHOOLAR)**	08 to 09
**Sample size (n)**	97	1112	81	25	702	121
**Girls (%)**	55.7	49.9	58.0	55.0	50.5	55.6
**Study Type**	Validity	Cross-sectional (no validity coefficient)	Validity	Validity	Cross-sectional (no validity coefficient)	Validity
**Suplements assessed**	Not declared	No	Yes	Yes	Not declared	Yes
**Nutritional database**	Willet	Standard Spanish Food Composition tables	General Intake Estimation System software -Denmark + USDA	Not declared	Not declared	USDA National Nutrient Database
**Blood Biomarkers**	b-Carotene, a-Carotene, b-Cryptoxanthin, a-tocopherol, ascorbic acid	α-Carotene, β-Carotene, Lycopene, Carotenes, Retinol, α-tocopherol, y-tocopherol, vitamin E	b-Carotene, a-Carotene, b-Cryptoxanthin, Lycopene, Lutein, Zeaxanthin	Total carotenoids, Lycopene, a-Carotene, b-Carotene, Lutein, Zeazanthin	β-Carotene, Lycopene, Tocopherol, Retinol	Total plasma carotenoids
**Biochemical Method**	HLPC (carotenoids, vitamin A and E) Spectrophotometry (vitamin C)	HPLC	HPLC	HPLC	Not declared	HPLC
**Fasting Time Length**	Not required	12 hour	Overnight fasting	10 hour overnight	Fasting (period not declared)	Non fasting

Supplementary Material 2 (Continued)

**Reference**	**Byers et al. (1993)**	**Royo-Bordonada et al. (2003)**	**Biltoft-Jensen et al. (2013)**	**Aguilar et al. (2014)**	**Augusto et al. (2014)**	**Medin et al. (2016)**
**Exposure assessment Method**	FFQ (111 food items)	FFQ (77 food items)	Web-based Dietary Assessment Software	Web-based 24h dietary recall and FFQ (27 food items)	FFQ	Web-based food recall
**Period**	Previous 3 months	Previous year	7 consecutive nights	24h recall: 3days FFQ: Previous week	Previous month	4 consecutive nights
**Report**	Parental	Mother	Children self-report	Parental	Interview with the mother	Parental
**Consumption**	Median Fruits and Vegetables (serving/day) Girls: 3.7 Boys: 2.9	Median Fruits and Vegetables 669 (g/d)	Mean fruits (jam, nuts, fruit syrup), juice and vegetables (g/d) 403 weekdays / 321 weekend	Median Fruits and Vegetables 3.0 (serving/day)	Never: n=100; vegetables ≥ 1 time/week: n=584; Fruits ≥ 4 times/week	Median fruits, juice and vegetables (g/d) 225.1
**Dietary Intake**	(b-carotene + a-carotene + cryptoxanthin mg/dl) Girls: 46; Boys: 37 (Retinol IU/dl) Girls: 41; Boys: 42	Diet Variety Index (together with other foods such as sausage)	b-Carotene + a-Carotene + b-Cryptoxanthin + Lycopene + Lutein þ zeaxanthin: 74 (mg/dl)	--	b-Carotene: 400 Lycopene:130 (nmol/l) Tocopherol:17.3 Retinol: 1.13 (μmol/l)	b-Carotene + a-Carotene + b-Cryptoxanthin + Lycopene + Lutein + zeaxanthin: 1.86 (mg/dl)
**Corr. Vit. A**	0.30; Retinol:0.25	0.13; Retinol: 0.08	0.58	0.08 (24h recall) 0.18 (FFQ)	Indicators of association (b coefficient)	0.47
**Corr. Vit. E**	0.24	0.10	--	--	129·6 (b coefficient)	--
**Corr. Vit. C**	0.34	--	--	--	--	--
**Criterion Validity Test**	Spearman	Pearson	Spearman	Multivariable linear regression	Pearson	Spearman
**Covariates in fully adjusted model**	Age, BMI, ethnicity, family history of artery disease, triglycerides, blood cholesterol, total caloric intake	Total caloric intake	Age, sex, BMI, parental education, total energy expenditure, fat intake and illness	Age, sex, race, BMI, weight	Age and sex	Age, sex, BMI, ethnicity, family structure, parental education
**Conflict of Interest**	Not declared	Not declared	None	NuSckin LLC	None	None
