# Supplementary material for: What is the Validity of Questionnaires Assessing Fruit and Vegetable Consumption in Children when Compared with Blood Biomarkers? A Meta-Analysis
Source: Nutrients. 2018 Oct 1;10(10):1396. doi: 10.3390/nu10101396 (PMC6212808; doi:10.3390/nu10101396)
Supplement: Supplementary file 1 [file nutrients-10-01396-s001.zip › Additional file 4_Exclusion.pdf]

Supplementary Material 4 Main reasons for exclusion of the full-text articles assessed for eligibility.

First Author	Public. Year	Article Title	Main reason for exclusion
Biltoft-Jensen et al.	2013	Evaluation of Web-based Dietary Assessment Software for Children: comparing reported fruit, juice and vegetable intakes with plasma carotenoid concentration and school lunch observations	Sample 11years
Byers, T.	1993	The accuracy of parental reports of their children's intake of fruits and vegetables: validation of a food frequency questionnaire with serum levels of carotenoids and vitamins C, A, and E	Included
Carlsen, M. H.	2011	Relative validity of fruit and vegetable intake estimated from an FFQ, using carotenoid and flavonoid biomarkers and the method of triads	Sample 18-80 years
Cullen, K. W.	2004	Validity and reliability of a behavior-based food coding system for measuring fruit, 100% fruit juice, vegetable, and sweetened beverage consumption: results from the Girls Health Enrichment Multisite Studies	Biomarker
Fiorentino, M.	2016	Nutrient intake is insufficient among senegalese urban school children and adolescents: Results from two 24 h recalls in state primary schools in Dakar	Sample 5-17 years
Fraser, G. E.	2016	Biomarkers of dietary intake are correlated with corresponding measures from repeated dietary recalls and food-frequency questionnaires in the Adventist Health Study-2	Sample ≥18 years
Golley, R. K.	2015	A dietary guideline adherence score is positively associated with dietary biomarkers but not lipid profile in healthy children	Sample (4-11) years
Greene, G. W.	2008	Correspondence of the NCI fruit and vegetable screener to repeat 24-H recalls and serum carotenoids in behavioral intervention trials	Sample ≥18 years
Knutsen, S. F.	2001	Comparing biological measurements of vitamin C, folate, alpha-tocopherol and carotene with 24-hour dietary recall information in nonhispanic blacks and whites	Sample ≥18 years
Medin, A. C.	2016	Associations between reported intakes of carotenoid-rich foods and concentrations of carotenoids in plasma: a validation study of a web-based food recall for children and adolescents	Included
Nguyen, L.	2013	Evaluating the relationship between fruit and vegetable intake using plasma and dermal biomarkers and reported dietary intake in 4th grade children	Sample 9-12 years
Royo-Bordonada, M. A.	2003	Food sources of nutrients in the diet of Spanish children: the Four Provinces Study	Biomarker
Royo-Bordonada, M. A.	2003	Greater dietary variety is associated with better biochemical nutritional status in Spanish children: The Four Provinces Study	Exposure
Royo-Bordonada, M. A.	2003	Spanish children's diet: compliance with nutrient and food intake guidelines	Biomarker
Ryman, T. K.	2015	Characterising the reproducibility and reliability of dietary patterns among Yup'ik Alaska Native people	Sample ≥18 years
Signorello, L. B.	2010	Biochemical validation of food frequency questionnaire-estimated carotenoid, α-tocopherol, and folate intakes among African Americans and non-hispanic whites in the southern community cohort study	Sample ≥18 years
Strauss, R. S.	1999	Comparison of serum concentrations of α-tocopherol and β-carotene in a cross-sectional sample of obese and nonobese children (NHANES III)	Sample (6-11); (12-18) years
Townsend, M. S.	2016	Vegetable behavioral tool demonstrates validity with My Plate vegetable cups and carotenoid and inflammatory biomarkers	No validity coefficient
Yin, S.	2000	Effect of green and yellow vegetables on serum carotenoid in children	Language (Chinese)
