# Supplementary figures and images for: What is the Validity of Questionnaires Assessing Fruit and Vegetable Consumption in Children when Compared with Blood Biomarkers? A Meta-Analysis
Source: Nutrients. 2018 Oct 1;10(10):1396. doi: 10.3390/nu10101396 (PMC6212808; doi:10.3390/nu10101396)

Funnel plot with pseudo 95% confidence limits

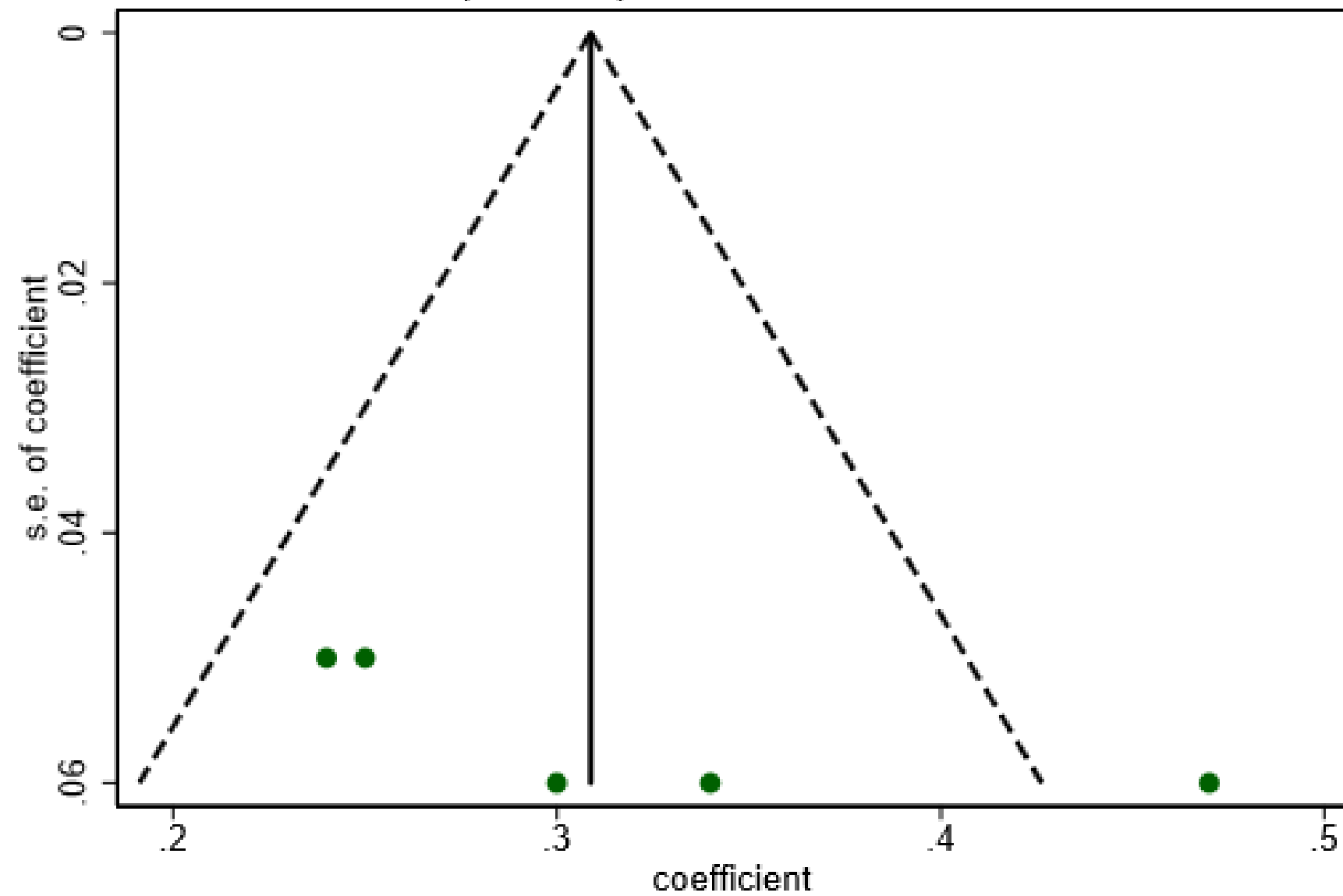

Supplement: Supplementary file 1 [file nutrients-10-01396-s001.zip › Additional file 3.pdf]
